# SCATrans: semantic cross-attention transformer for drug–drug interaction predication through multimodal biomedical data

**DOI:** 10.1186/s12859-025-06165-6

**Published:** 2025-06-10

**Authors:** Shanwen Zhang, Changqing Yu, Chuanlei Zhang

**Affiliations:** 1 School of Electronic Information, Xijing University, Xi’an, 710123 China; 2 School of Artificial Intelligence, Tianjin University of Science and Technology, Tianjin, 300457 China

**Keywords:** Drug-drug interaction (DDI), DDI predication (DDIP), Multimodal feature fusion (MMFF), Semantic cross attention transformer (SCAT)

## Abstract

Predicting potential drug-drug interactions (DDIs) from biomedical data plays a critical role in drug therapy, drug development, drug regulation, and public health. However, it remains challenging due to the large number of possible drug combinations, and multimodal biomedical data, which is disorder, imbalanced, more prone to linguistic errors, and difficult to label. A Semantic Cross-Attention Transformer (SCAT) model is constructed to address the above challenge. In the model, BioBERT, Doc2Vec and graph convolutional network are utilized to embed the multimodal biomedical data into vector representation, BiGRU is adopted to capture contextual dependencies in both forward and backward directions, Cross-Attention is employed to integrate the extracted features and explicitly model dependencies between them, and a feature-joint classifier is adopted to implement DDI predication (DDIP). The experiment results on the DDIExtraction-2013 dataset demonstrate that SCAT outperforms the state-of-the-art DDIP approaches. SCAT expands the application of multimodal deep learning in the field of multimodal DDIP, and can be applied to drug regulation systems to predict novel DDIs and DDI-related events.

## Introduction

With the increasing number of new drugs and new diseases, and the growing prevalence of compound formulations on the market, drug combination therapy (DCT) is becoming more and more popular and necessary to treat patients with complex diseases and co-diseases [1]. Over the past 10 years, the use of more than 2 drugs has increased from 25.4 to 41.2%, the use of more than 5 drugs has increased from 6.3 to 14.7%, and more than 40% of Americans over the age of 65 take 5 or more drugs to treat multiple conditions. However, DCT easily leads to DDIs, including unwanted DDIs, side effects, and adverse drug reactions (ADRs) with serious consequences such as fever, diarrhea, headache, nausea, and even death. For example, the combination of Acetazolamide with Insulin can reduce hypoglycemic reactions, while the combination with Adrenocorticotropin, Glucocorticoids, especially Corticosteroids can cause severe hypokalemia. With the increasing use of DCTs, ADRs caused by DCT are becoming more serious [2]. The probability of ADRs occurring when taking 6 to 10 drugs at the same time is 7%, and the probability of ADRs occurring when taking 10 to 20 drugs at the same time is more than 40%, especially 20% of the elderly who take more than 10 drugs at the same time [3]. Therefore, predicting DDI in advance is crucial in clinical practice. It helps reduce the probability of ADRs, and optimizes the drug development and drug-marketing regulatory process. The Weekly Adverse Drug Reaction Report is a one-stop shop for ADR needs, providing the latest global ADR news, including label changes, discontinuation due to safety concerns, ADR studies and current drug safety issues (https://link.springer.com/journal/40278). Its content comes from media releases, journals, scientific conferences, regulatory agency websites and announcements from national regulatory agencies. There are various DDI datasets and DDIP systems, such as DDIExtraction2013, DrugBank 4.0, PharmGKB, DrugCentral, DDInter [4–6], where DDIExtraction2013 and DrugBank 4.0 are two widely used multimodal DDI datasets, including drug sentence, drug targets, drug description, drug molecular graph chemical structures (SMILES), enzymes and pathways. The advantage of multimodal DDIP is that it overcomes the limitations of a single data source by integrating multimodal information, comprehensively improving the accuracy and interpretability of DDIP [7]. For example, chemical structures can reveal reaction potential at the molecular level, target data can identify enzyme competition or pathway crossing, and clinical records provide real-world evidence of DDIs. This multimodal data fusion not only captures more complex mechanisms of DDI, but also reduces false positives/negatives and provides mechanistic explanations for medical decisions. The multimodal method is especially suitable for the multi-drug combination scenario and provides a reliable basis for personalized medication and drug safety monitoring.

Due to the difficulty, high cost, and lengthy time required for experimental and handcrafted DDIP, the number of known DDIs is limited, and it is difficult to keep up to date DDIs. As a result, a great deal of DDI information remains hidden in the vast biomedical literature, including journal papers, technical reports, ADR event reports and media corpus, making up an extremely rich and growing source of DDI information [8, 9]. Predicting potential DDIs from biomedical data can reduce unexpected DDI, DDI-related costs, and health threats. It is a good complement to existing DDI datasets, but it remains an important and challenging work, due to the irregularity, fuzziness and redundancy of the multimodal biomedical data [10–12].

Deep Learning (DL) and Transformer, and their improved models and combinations are specialized in extracting high-level drug features, and have been widely applied to DDIP task, with remarkable results. However, many DDIP models cannot extract enough effective global and local context features. Inspired by the promising performance of MFD-GDrug [10], SRR-DDI [11] and TransGRU [12] to extract the local discriminant features and capture the global dependencies, a Semantic Cross-Attention Transformer (SCAT) model is constructed for DDIP. Different from the existing DDIP methods, it makes use of multimodal biomedical data, BiGRU, and Cross-Attention to extract the local–global context semantic feature for multimodal DDIP. The three main contributions of this work are summarized as follows:

- The multimodal data associated with DDIP are complementary, and their effective combination contributes significantly to DDIP.
- The Cross-Attention module is used to take into account the different contributions and correlations of various drug features of the drugs, better retaining the intrinsic characteristics of the drugs while predicting their DDIs.
- The extensive experiment results demonstrate that SCAT achieves superior DDIP performance compared to the state-of-the-art methods.

The remaining of this paper is organized as follows: Sect. "Related work" briefly introduces the related work. SCAT and its main components are described in detail in Sect. "Method". A number of comparative experiments and result analysis are presented in Sect. "Experiments", and finally the paper is summarized in Sect. "Conclusion".

## Related work

DDIP through clinical trials are expensive, time-consuming, and even unfeasible when facing large-scale data and experimental conditions. With the rapid development of computational science and the increasing popularity of interdisciplinary research, many DDIP methods have been proposed in recent years, which are roughly divided into four categories: Machine Learning(ML)-based [13, 14], DL-based [8, 15], Transformer-based [11, 16], and multimodal-based [10, 12] methods.

### ML-based methods

To comprehensively understand DDIP, Wang et al.[14] systematically reviewed DDIP research from three aspects: (1) Classical DDI dataset, including drug dataset, side effect dataset and DDI information dataset, (2) Commonly used drug properties, focusing on chemical, biological and phenotypic properties to represent drugs, (3) Popular ML methods, such as DDIP methods based on shallow learning, DL, recommendation system and knowledge graph. They summarized and compared the relevant studies, the research status of ML-based DDIP approaches and the existing problems, and pointed out future challenges, potential opportunities and future research directions. Han et al.[17] reviewed the progress of ML in predicting unknown DDIs, introduced commonly used datasets, briefly described each approach, summarized the advantages and disadvantages of DDIP models, finally pointed out the challenges and prospects of ML-based DDIP methods.

From the above analysis, it is seen that most of ML-based methods rely on the drug similarity assumption, and their results depend on the extracted manual features, which leads to low generalization ability.

### DL-based methods

Since DL has performed well in the fields of computer vision and natural language processing (NLP) [18], most of DDIP methods are based on various DL models. These methods do not require to manually define features, using a multi-scale convolution window to extract the context information of DDI, resulting in great generalization ability. Wu et al.[19] adopted stacked bidirectional gated recurrent unit (SBGRU) and CNN to extract lexical information and entity location information, respectively, and used an attention pooling layer to improve DDIP performance by assigning weights to each word feature. Zhong et al.[20] developed a DDI-GCN model for DDIP based on chemical structure and graph Convolutional networks (GCN), achieved state-of-the-art predictive performance on independent retention sets. The model provides a visualization of the structural features associated with DDI, which can be used to investigate the underlying mechanisms. Dou et al. [21] reviewed the state-of-the-art literature related to DDI extraction from the deep neural network perspective, introduced the general process of DDIP based on DL for comprehensive analysis, summarized and analyzed the various feature supplement methods and their merits and demerits, compared all the feature supplement methods, and gave some suggestions to the current DDIP problems and future research directions. Du et al.[22] proposed a self-supervised multi-view graph representation learning (SMG-DDI) for DDIP. It adopts a self-supervised approach, leveraging the large, unlabeled molecular dataset ZINC15 to extract molecular features, utilizes a pre-trained GCN to generate molecular graph representations between views, with atoms as nodes and chemical bonds as edges, captures the interactions between molecules in view, embeds the final DDI in-view analysis. Luo et al.[15] introduced the existing DDIP methods, divided them into three broad categories: DL-based methods, KG-based methods, and hybrid methods, compared these methods on a baseline dataset, and briefly discussed the challenges associated with DDIP, including asymmetric DDIP and higher-order DDIP.

From the above analysis, it is seen that most existing DL models can effectively learn rich local discriminant features from a large number of training samples, and outperform various feature-based ML methods, but their ability to capture global features is limited.

### Transformer-based methods

Transformer is a typically stacked DL model with multiple layers of the same encoder and decoder [23]. It has been widely applied to various fields, such as natural language processing (NLP), computer vision and DDIP, and achieved remarkable success [24, 25]. It uses Self-Attention (SA) to capture contextual relationships within sequential data. Unlike traditional CNN and its variants, it assigns different attention weights according to different parts of the input sequence, thus better capturing semantic relationships [16]. It extends SA to multi-head attention (MHA) to learn different attention weights and better capture different types of relationships, and process different information subspaces in parallel. Residual connections and layer normalization are adopted to reduce the problems of gradient disappearance and explosion during training, and make the model easier to train [26]. Chen et al. [16] proposed a dual-attention graph Transformer framework for DDIP. It integrates short-term and long-term dependencies within drug molecules to identify the key local structures, and utilizes graph contrastive learning to maximize the similarity of representations between different views, thereby better recognizing molecular structures. Zaikis et al.[27] proposed an end-to-end pipeline DDIP approach with Transformer and pre-training weights. By using BioBERT pre-training weights to integrate prior knowledge, the method outperforms the current state-of-the-art methods on the DDI Extraction-2013 corpus in both drugs named entity recognition and overall DDIP tasks.

From the above analysis, it is seen that Transformer is powerful in capturing context features between two sequences, but its local feature extraction capability is limited.

### Multimodal-based methods

It is validated that the multi-features of drug targets, enzymes, pathways, and chemical substructures can greatly improve the DDIP performance of the models [7]. Deng et al.[28] proposed a deep multimodal feature fusion framework to learn the cross-modal representation of drug pairs, and predict DDI by integrating drug molecular graphs, pharmacochemical substructures, drug enzymes, and pathway features. Asada et al. [29] proposed a deep multimodal DDIP approach utilizing large-scale plain text, drug description, and molecular structure. The results show that DDIP can be improved by combining multimodal data. Zhang et al.[30] proposed a multimodal DDIP algorithm by extracting the feature interactions from drug classes, targets, pathways, and enzymes as feature vectors, using Jaccard similarity as a measure of drug similarity, and constructing CNN as a predictor of DDI. Gan et al.[31] proposed a deep multimodal feature fusion framework (DMFDDI) for DDIP, which fuses the features of DDI network, drug molecular graph and biochemical similarity to predict DDIs. It consists of multimodal feature extraction, feature fusion and DDI predictor, where 3 feature extraction modules are used to learn low-dimensional embedding vectors of different drug features, a multimodal feature fusion module is used to fuse three aspects of drug feature embedding, and the fused feature vector is input into the predictor to predict DDI. Shi et al.[32] proposed a subgraph enhance model for DDIP. They used the knowledge subgraph information of drugs to achieve large-scale plain text prediction without a lot of annotation, and predicted the specific type of DDI for each drug pair. Asfand et al.[33] constructed a multimodal CNN-DD to predict DDI events. The results on various iterations of drug features in different sets show that the feature set of chemical substructures, biomedical texts, targets and enzymes can improve DDIP performance better than other feature sets in predicting DDI events.

From the above analysis, it is obvious that the single feature extraction method cannot fully characterize DDIP information, using multimodal biomedical data is more informative and efficient than using single data, and the multimodal DDIP methods are superior to other existing DDIP approaches. However, there are some limitations. For example, the existing methods are mainly based on biology and chemistry, and rarely pay attention to the potential correlation between other multimodal features and DDI features, while ignoring the different importance of multiple drug features for DDIP. To improve the DDIP performance, a Semantic Cross-Attention Transformer (SCAT) model is constructed for DDIP through multimodal biomedical data. It can effectively capture the interaction features between multimodal medical data, improve the DDIP performance in cross-domain DDIP tasks and enhance the generalization performance.

## Method

**Fig. 1:**
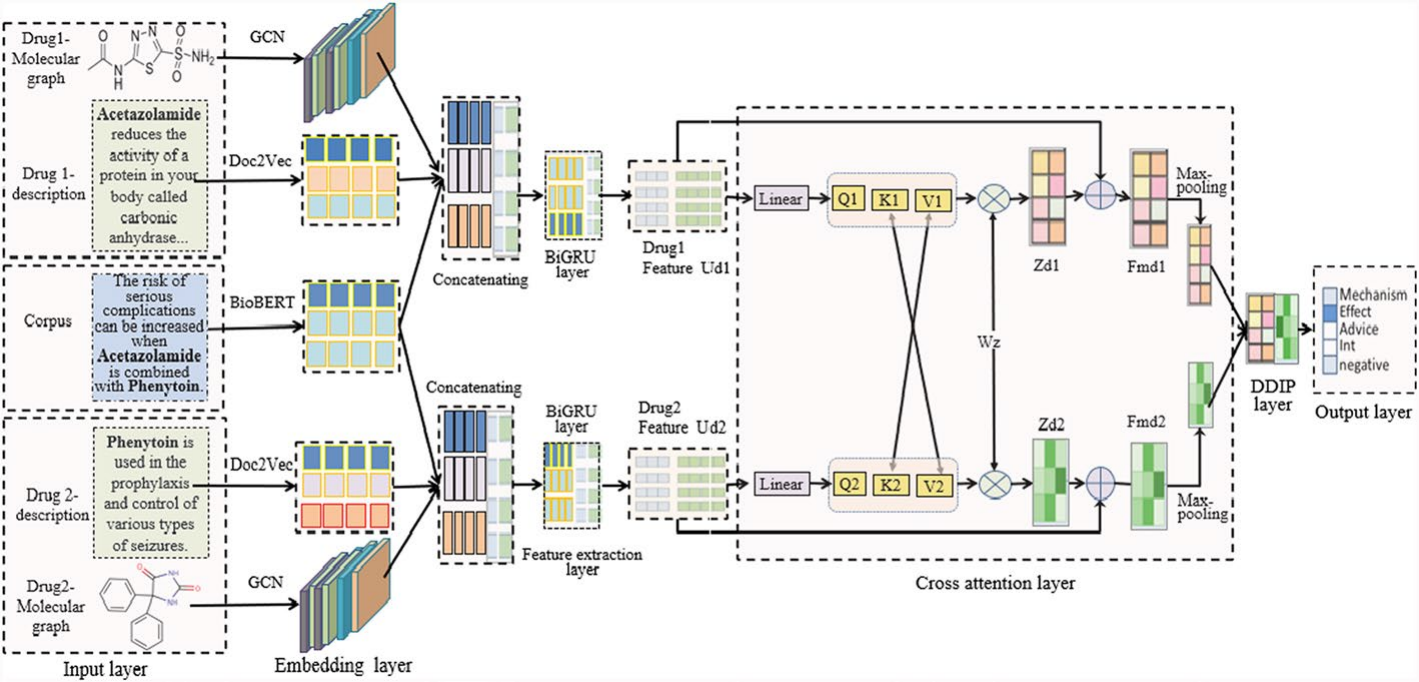
The architecture of SCAT

The architecture of SCAT is illustrated in Fig. 1, consisting of six layers: Input layer, Embedding layer, Feature extraction layer, Cross-Attention layer, DDIP layer, and Output layer. The input data of SCAT are drug sentence, drug description and drug molecular graph.

Given a sentence of biomedical corpus $S_{w} = [wor_{1} ,wor_{2} ,...,wor_{n} ]$ with *n* ≥ 2 drugs, there are $\left(\genfrac{}{}{0pt}{}{\text{n}}{2}\right)$ possible drug pairs. Only the pair explicitly annotated (or contextually dominant) is designated as the target interaction. In DDIP task, the target drug pair is replaced with tokens drug1 and drug2 in sentence order, while the other drugs are replaced with tokens drug0, and all punctuation marks and some meaningless stop-words are removed to overcome overfitting problem [17, 18]. The drug description sentences of drug1 and drug2 are noted as $Sm_{1}$ and $Sm_{2}$, and corresponding drug molecular graphs are denoted as *G*_1_ = (*V*_1_, *E*_1_) and *G*_2_ = (*V*_2_, *E*_2_), where *V*_*i*_ and *E*_*i*_ are the atom set and edge set (*i* = 1,2), respectively. The main steps of SCAT based DDIP method are introduced as follows.

### Multimodal data embedding and concatenating

BioBERT, Doc2Vec and Graph2Vec are adopted to embed the sentence *Sw* of biomedical corpus, two drug description sentences $Sm_{1}$ and $Sm_{2}$, and two drug molecular graphs G1 and G2 into a real-valued low-dimensional vectors, respectively, and concatenated as two embedding vectors of drug1 and drug2.

#### Biomedical word vector embedding.

Word2Vc, GloVe, ELMo, CoVe and BERT are 5 widely used word embedding models. Word2Vc and GloVe focus on learning context-free word representations, Word2Vec is a "predictive" model, while GloVe is a "count-based" model. ELMo uses a two-way language model, CoVe uses machine translation to embed contextual information into word representations, and BERT is a pre-trained model by combining information from two-way representations, rather than one-way representations, which is essential for representing words in natural language. BioBERT(Bidirectional Encoder Representations from Transformers for Biomedical Text Mining) is an improved BERT model with the same structure as BERT, but significantly outperforms BERT and the state-of-the-art models on many biomedical text mining task[34]. Due to minimal architectural modifications, it is widely applied to biomedical text mining. It can be freely pretrained the training weights in https://github.com/naver/biobert--pretrained, and the source code for fine-tuning BioBERT can be obtained at https://github.com/dmis-lab/biobert.

The preprocessed sentence *Sw* of biomedical corpus is input into BioBERT to generate word vector embedding of each word, and concatenated as the sentence vector, *Ew* is tokenized and encoded using the WordPiece tokenizer, these tokens are enriched with token embeddings, positional embeddings, and segment embeddings, and represented in the model as

1 $$ Ew = [w_{{[_{CLS} ]}} ,w_{1} ,w_{2} ,...,w_{n} ,w_{[SEP]} ] $$

where *w*_*i*_ is the embedding for each token, $w_{{[_{CLS} ]}}$ is placed in the first sentence, and $w_{[SEP]}$ is used to separate two input sentences.

The embedded sequence is processed through multi-layer bidirectional Transformer encoder. Each layer employs a multi-head self-attention (MHSA) to capture the dependencies and relationships between words in *Sw*. MHSA is defined as:

2 $$ Atten(Q,K,V) = Soft\max (QK^{T} /\sqrt {d_{k} } )V $$

where* Q*,* K* and* V* are the query, key, and value matrices, respectively, and $d_{k}$ is a scaling factor.

By Eq. (2), the context-attention embeddings $Es = [e_{{[_{CLS} ]}} ,e_{1} ,e_{2} ,...,e_{n} ,e_{[SEP]} ]$ is generated to capture the semantic and relational information of words within the entire sentence. The embedding is particularly effective for DDIP task. To make the output vector more generalized, the average output of the last 4 layers of BioBERT model is used to get the embedding vectors of the sentence tokens, also named *Es*.

#### Drug description vector embedding.

**Fig. 2:**
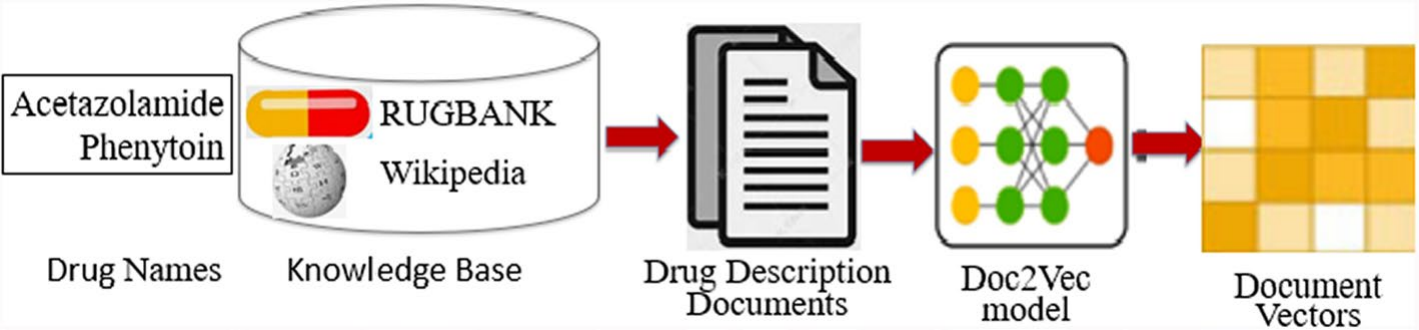
The embedding process of drug description documents by Doc2Vec

Word2Vec and Doc2Vec are both neural network-based embedding models that are widely used to convert text data into vector representations. But there is a difference between them, Word2Vec is a word vector learning model, mainly for word embedding, trained on individual words. Doc2Vec is a derivative of Word2Vec, mainly used for the classification of sentences, is trained on variable length text. With Word2Vec, words can be predicted based on context, while Doc2vec can be used to measure relationships between complete documents. The drug description sentences $Sm_{1}$ and $Sm_{2}$ of drug1 and drug2 can be obtained through DrugBank and Web-crawler on Wikipedia, and Doc2Vec is employed to transform the drug description document into a vector representation, denoted as $Ed_{1}$ and $Ed_{2}$. The description vectors $Ed_{1}$ and $Ed_{2}$ are used as external knowledge of two drugs to improve the context representation of the sentence. Figure 2 describes the embedding process of drug description documents with Doc2Vec, where Acetazolamide and Phenytoin are two drug entities. The document vectors are used as the drug description information in Fig. 1.

#### Molecule graph vector embedding.

**Fig. 3:**
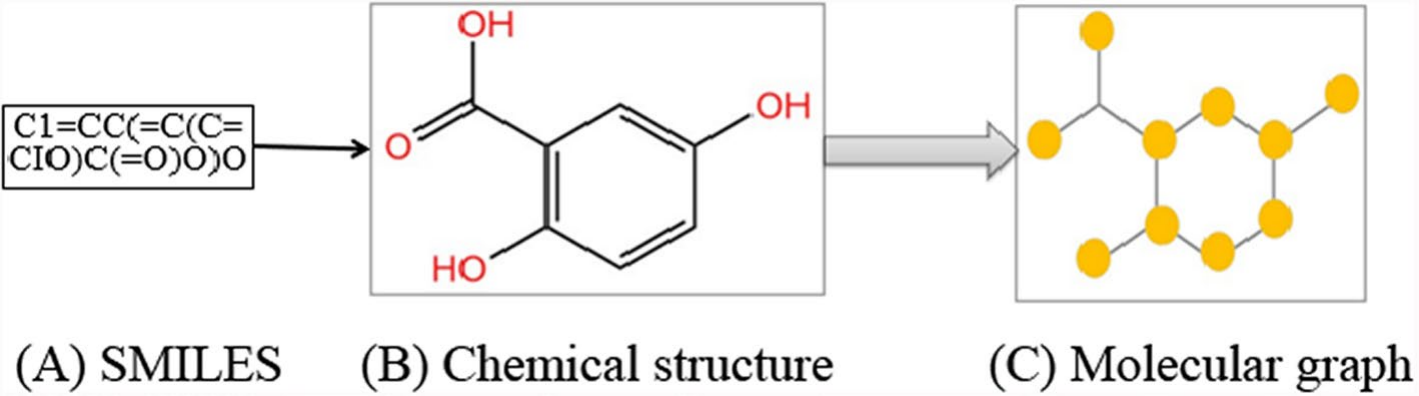
Preprocessed the drug SMILES into Molecular graph

Simplified molecular input line entry specification (SMILES), chemical structure and molecular graph are three common drug molecular representation formats, which can be applied in DDIP. These can be converted into each other, as shown in Fig. 3. A molecular graph is a special type of undirected graph, where nodes and edges are atoms and bonds, respectively. Graph2Vec (https://github.com/benedekrozemberczki/graph2vec) is an open-source graph embedding model based on TensorFlow. It provides an effective vectorization representation for graph to process and analyze graph data by using DL model. It is used to convert each molecule graph of drug1 and drug2 into a fixed-length vector $Eg_{1}$ and $Eg_{2}$, respectively [20].

#### Feature concatenating.

The above three kinds of embedding vectors of drug1 and drug2 are concatenated to obtain two embedding vectors as follows,

3 $$ \begin{gathered} E_{1} = [Es;Ed_{1} ;Eg_{1} ] \hfill \\ E_{2} = [Es;Ed_{2} ;Eg_{2} ] \hfill \\ \end{gathered} $$

$E_{1}$ and $E_{2}$ are two comprehensive vectors of drug1 and drug2, and are input into BiGRU (Bidirectional Gated Recurrent Unit) to further capture the local–global features and contextual representation of the multimodal data, respectively.

### Feature extraction

GRU is made up of a reset and an update gates to determine the output of the gated loop unit. Both control mechanisms can retain long-sequence information that is not deleted over time and are not irrelevant to DDIP. BiGRU consists of two GRUs to encode $E_{1}$ and $E_{2}$ forward and backward, respectively, learning contextual semantic features, and the output of BiGRU are as follows,

4 $$ Ud_{i}^{t} { = [}\overrightarrow {{G_{i}^{t} }} \oplus \overleftarrow {{G_{i}^{t} }} ] $$

where* i* = 1,2, ‘⊕’ is concatenating operation, $\overrightarrow {{G_{i} }} {\text{ = GRU(}}E_{i}^{t} {,}\overrightarrow {{G_{i}^{t - 1} }} {)}$ and $\overleftarrow {{G_{i} }} {\text{ = GRU(}}E_{i}^{t} {,}\overleftarrow {{G_{i}^{t - 1} }} {)}$ are the output of bidirectional GRUs at the time *t*, respectively, $E_{i}^{0} = E_{i}^{{}}$ calculated by Eq. (3).

From Eq. (4), the output vectors of drug1 and drug2 can be generated as, $Ud_{i}^{{}} { = [}Ud_{i}^{1} ,Ud_{i}^{2} ,...,Ud_{i}^{Gd} ]$, where* i* = 1,2, *Gd* is the dimensionality of feature vector. $Ud_{1} ,Ud_{2}$ are input into Cross-Attention layer.

### Cross-attention

Cross-Attention is widely used in the Transformer architecture to combine two different embedding sequences to capture the dependencies between them. Unlike Self-Attention, its inputs come from different sequences, one for Query and the other for Key and Value, as shown in Fig. 1. In SCAT, Cross-Attention dynamically focuses on different parts of multimodal data depending on the DDIP task. It can handle the semantic relationship between drug-drug pair feature sequences, using MHSA to obtain multi-set attention results. Given two feature vectors $Ud_{1}$ and $Ud_{2}$ of drug1 and drug2, Cross-Attention is implemented by calculating the correlation between each element in one vector and all elements in the other vector, and weighting each element in both vectors according to the correlation. The main steps are listed as follows,

For drug1 and drug2, the embedding vectors $Ud_{i} (i = 1,2)$ are passed through the linear layer to calculate the drug query-vector, drug key-vector and drug value-vector as follows:

5 $$ \begin{gathered} Q_{d1}^{j} = Ud_{1} \cdot W_{q}^{j} ,\;\;K_{d1}^{j} = Ud_{2} \cdot W_{k}^{j} ,\;\;V_{d1}^{j} = Ud_{2} \cdot W_{v}^{j} \hfill \\ Q_{d2}^{j} = Ud_{2} \cdot W_{q}^{j} ,\;\;K_{d2}^{j} = Ud_{1} \cdot W_{k}^{j} ,\;\;V_{d2}^{j} = Ud_{1} \cdot W_{v}^{j} \hfill \\ \end{gathered} $$

where $W_{q}^{j} ,\;W_{k}^{j} ,\;W_{v}^{j}$ are three weight matrices in the linear layer, *j* = 1, 2,···, *h*, where *h* is the number of attention heads.

In Eq. (5), two drug feature representations share the same weights. Through Softmax function, two drug attention matrices are computed as,

6 $$ \begin{gathered} A_{d1}^{j} = Soft\max (Q_{d1}^{j} \cdot K_{d1}^{j} /\sqrt {d_{{K_{d1}^{j} }} } ) \hfill \\ A_{d2}^{j} = Soft\max (Q_{d2}^{j} \cdot K_{d2}^{j} /\sqrt {d_{{K_{d2}^{j} }} } ) \hfill \\ \end{gathered} $$

where $d_{{K_{d1}^{j} }} = d_{{K_{d2}^{j} }} = d_{head}$ is the matrix dimension for each drug.

The drug attention matrix of each attention head is multiplied by the corresponding drug-value matrix to obtain the drug feature map for each attention head. The drug feature maps of all attention heads are concatenated along the channel dimension and fed into the linear layer to obtain the final drug feature representation as follows,

7 $$ \begin{gathered} Z_{d1}^{{}} = Concat(A_{d1}^{j} \times V_{d1}^{j} ) \times W_{Z}^{{}} \hfill \\ Z_{d2}^{{}} = Concat(A_{d2}^{j} \times V_{d2}^{j} ) \times W_{Z}^{{}} \hfill \\ \end{gathered} $$

where $W_{Z}^{{}} \in R^{{u_{qd1} \times u_{qd2} }}$ is the shared weight matrix,* j* = 1, 2, ···, *h*.

The interested feature maps are combined with the original feature maps to obtain the final drug feature maps as follows,

8 $$ \begin{gathered} F_{zd1}^{{}} = (Z_{d1}^{{}} + U_{d1}^{{}} )/2 \hfill \\ F_{zd2}^{{}} = (Z_{d2}^{{}} + U_{d2}^{{}} )/2 \hfill \\ \end{gathered} $$

where $U_{d1}^{{}} ,U_{d2}^{{}}$ are the embedding vectors of drug1 and drug2 calculated by BiGRU.

Through global max-pooling operation, two fixed-size feature vectors are obtained as follows,

9 $$ \begin{gathered} F_{md1}^{{}} = \max {\text{pooling}}(F_{zd1}^{{}} ) \hfill \\ F_{md2}^{{}} = \max {\text{pooling}}(F_{zd2}^{{}} ) \hfill \\ \end{gathered} $$

where $F_{md1}^{{}}$ and $F_{md2}^{{}}$ are two *D*-dimensional vectors of two drugs, *D* is the dimensions of the comprehensive vectors.

### DDIP

DDIP can be regarded as either binary or multi-class classification depending on the application scenario, this work specifically addresses multi-class classification to distinguish between different types of DDIs. DDIP is considered as a multi-class classification problem. Given a biomedical corpus with a drug entity pair, in a biomedical corpus, the goal of the DDIP task is to predict the DDI between the entity pair from all positive DDIs types (int, effect, advice, mechanism) and a “negative” type.

According to Eq. (9), the DDI representations are learned from $F_{md1}^{{}}$ and $F_{md2}^{{}}$ of drug1 and drug2, the attention weight $\hat{a}_{12}$ is calculated as follows,

10 $$ \widehat{a}_{12} = W^{T} {\text{ReLU(}}W_{12} [F_{md1}^{{}} ,F_{md2}^{{}} ] + b) $$

where $W$,$W_{12}^{{}} ,b$ are the trainable weight vector, matrix and bias evaluated from fully CNN, $[F_{md1}^{{}} ,F_{md2}^{{}} ]$ is concatenation of $F_{md1}^{{}}$ and $F_{md2}^{{}}$.

$\hat{a}_{12}$ is activated by Softmax function as follows,

11 $$ a_{12}^{d} = \exp (\hat{a}_{12}^{d} )/\sum\limits_{i = 1}^{D} {\exp (\hat{a}_{12}^{i} )} $$

where $a_{12}^{d}$ is the* d*-th attention weight of $a_{12}$, $\hat{a}_{12}^{d}$ is the *d*-th attention weight of $\hat{a}_{12}$(*d* = 1,2,…, *D*).

Then a DDI link embedding vector $F_{12}^{{}}$ is obtained as follows,

12 $$ F_{12}^{{}} = a_{12}^{{}} *(F_{md1}^{{}} \otimes F_{md2}^{{}} ) $$

where $\otimes$ is the element-wise product, $a_{12}^{{}} = [a_{12}^{1} ,a_{12}^{2} ,...,a_{12}^{D} ]$ is a *D*-dimensional attention vector to capture the different importance in $F_{md1}^{{}} \otimes F_{md2}^{{}}$.

Finally, a softmax layer is to used obtain the probability distribution over all DDI classes,

13 $$ p\, = \,{\text{Softmax }}(F_{12} ) $$

### Model training

SCAT is optimized by adopting the cross-entropy loss function to concurrently optimize the learnable parameters and Adam optimizer with default parameters. Between hidden layers, batch normalization is utilized to accelerate the model convergence, and dropout is used to avoid overfitting and improve generalization performance. The cross-entropy loss is defined as follows,

14 $$ Loss = - \sum\limits_{i = 1}^{M} {[L_{i} } \log p(L_{i} |D_{i} ) + (1 - L_{i} )*\log (1 - p(L_{i} |D_{i} ))] $$

where *M* is the number of training drug-pairs,* D*_*i*_ is the *i*th drug-pair,$L_{i}$ is the actual label value of *D*_*i*_, $p(L_{i} |D_{i} )$ is the predicted probability of *D*_*i*_, and $p(L_{i} |D_{i} )$ is calculated by Eq. (13).

## Experiments

The SCAT based DDIP method is validated and compared with five state-of-the-art DDIP methods: MFD-GDrug [12], DrugDAGT [16], DDI-GCN [20], DDI-MuG [35], DGNN-DDI [36] and Graph Mutual Interaction Attention (GMIA) [37]. All models are implemented with the same experiment set. They are simply introduced as follows.

To solve this problem, we propose MFD-GDrug, a multimodal deep learning model. Using ESM pre-training model, we extract protein features and use CNN to represent protein features. For drugs, we integrate multi-modal features of drug molecular structure, including 3D features extracted from Mol2vec and topological information of drug diagram structure extracted through graph convolutional neural networks (GCN). By combining structural characterization and pre-trained embedding, our model effectively captures GPCR-drug interactions.

DrugDAGT is a dual-attention graph Transformer model for DDIP. It uses graph Transformer to capture short-distance and long-distance dependencies, and adopts graph contrastive learning to better represent molecular structures.

DDI-GCN is a attention GCN model to predict DDI using drug chemical structures.

DDI-MuG is a multi-aspect graph-based DDI extraction model by using two graphs to get syntactic features from input instance and word co-occurrence information within the entire corpus, respectively.

DGNN-DDI is a dual graph neural network (GNN) to learn drug molecular features using molecular structure and interactions.

GMIA is a Graph learning framework to predict DDI by efficiently representing drug molecules. It can capture the mutual interaction context between two molecular graphs in a drug pair.

The data sources for these models come from the same DDIExtraction-2013 dataset, but are not the same from each other, as the above analysis.

### DDIExtraction2013 dataset

**Fig. 4:**
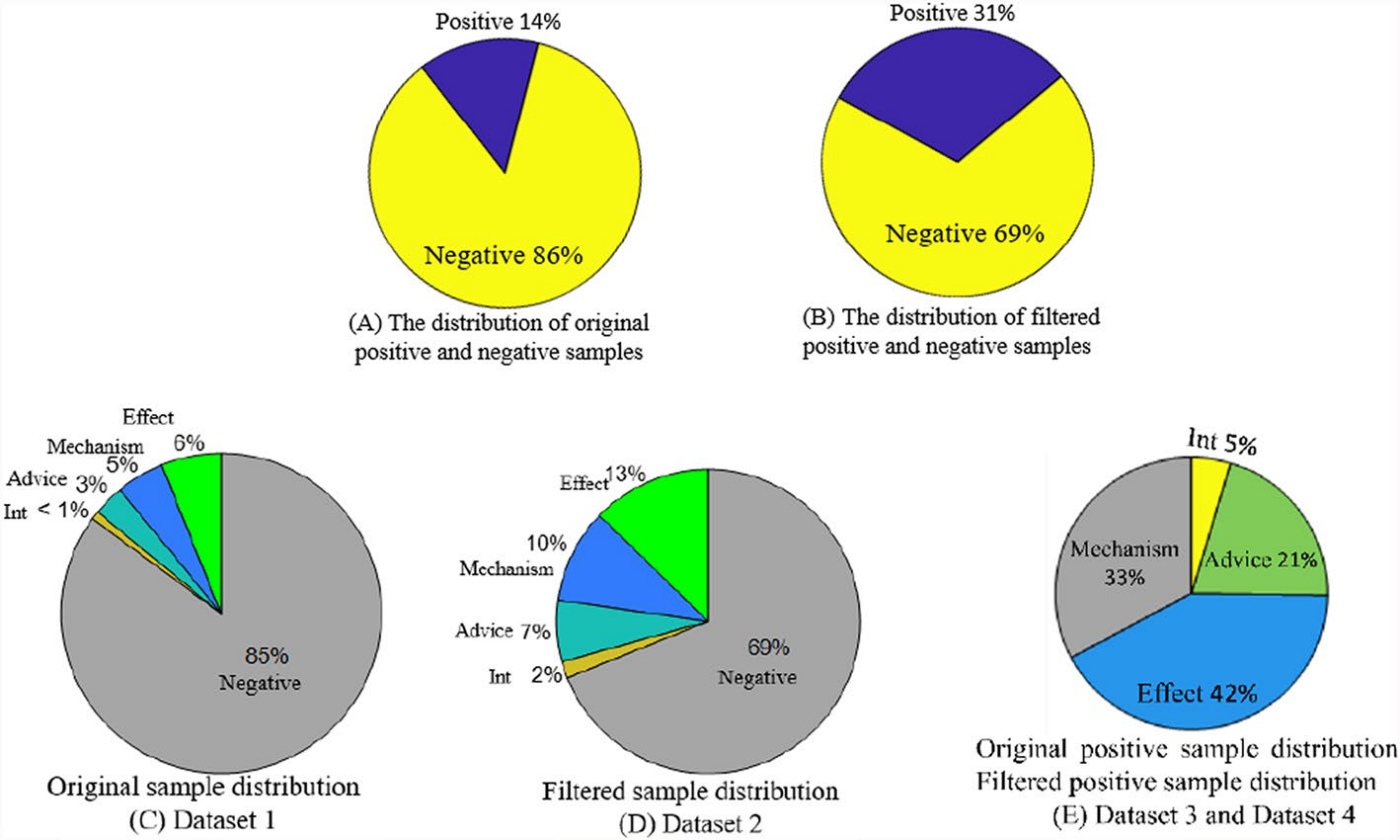
Sample distribution of DDIExtraction2013 dataset

DDIExtraction2013 (http://labda.inf.uc3m.es/ddicorpus) is a challenging annotated-sentence dataset, widely used to validate various DDIP methods. It integrates bioinformatics and chemical informatics resources to provide detailed drug data, including drug chemical substructures, enzymes, targets, pathways and DDIs. It consists of 175 abstracts from MedLine and 730 articles from DrugBank. All abstracts are classified into five categories based on clinical outcomes: Negative, Effect, Mechanism, Advice and Int, where Negative means that two drugs are unrelated, and Adverse refers to side effects or adverse reactions between two drugs. The detailed information and distribution of this dataset are described in Table 1 and Fig. 4. From Table 1 and Fig. 4, it is seen that the dataset is extremely uneven and imbalanced, and contains a large number of negative instances over 100 times that of Int instances, which makes the model difficult to predict the small-class ‘Int’ with fewer instances. To alleviate this problem and improve DDIP performance, two rules are adopted to remove the possible negative instances when two drugs have the same name, and one drug name is a special case or abbreviation of another drug name in a drug pair [20–22]. The detailed information of the filtered dataset is also shown in Table 1 and Fig. 4.

**Table 1:** The detail statistics of DDIExtraction2013 dataset

Sample number	Type	Original		Filtered	
		Training set	Test set	Training set	Test set
Positive	Effect mechanism advice int	1685	360	1675	359
		1318	302	1309	301
		826	221	824	221
		189	96	188	96
	Total	4018	979	3996	977
Negative		23,756	4737	8987	2049
Total		27,774	5716	12,983	3026

Four datasets are constructed to test the performance of SCAT, as shown in Table 2. Their sample distributions are shown in Fig. 4, respectively, where Fig. 4A and B show the distribution proportions of positive and negative samples in original and filtered datasets, Fig. 4C and D show the distribution proportions of five DDI types in original and filtered datasets, and the distribution proportions of positive samples are the same before and after filtering, as shown in Fig. 4E.

**Table 2:** Four datasets

Dataset name	Sample composition
dataset-1	All original samples
dataset-2	All filtered samples
dataset-3	All original samples without negative samples
dataset-4	All filtered samples without negative samples

From Table 1, it is seen a large number of negative instances are filtered, while a small number of positive instances are also filtered. In datasets 2 and 4, these filtered positive instances are deleted, since these instances can degrade network performance. From Table 1 and Fig. 4, it is seen that the four datasets are also uneven.

The model performance can be improved significantly through data preprocessing, including extracting named entities, labeling and anonymizing target drugs, converting all words to lowercase letters, replacing all numbers with a special label "drug" with a regular expression, each drug pair in the medical corpus representing a possible instance of DDI, replacing the two drugs mentioned in each instance with drug1 and drug2, while the rest of the drugs with drug0 [3, 18, 22].

### Experiment setup

DDIP experiments are conducted on the training-test sample distribution given in Table 1 and by fivefold cross validation (5FCV) on four datasets in Table 2, where the known DDIs in the training set are used to train the DDIP models and the unknown DDIs in the test set are used to measure the DDIP performance. All models are implemented using Python 3.8 and Pytorch 1.13.0, Ubuntu OS with a NAVID GeForce TRX 3090 GPU. Four metrics Accuracy (Acc), precision (P), recall (R), and F1-score are adopted to measure the performance of DDIP models, calculated by

15 $$ \begin{gathered} Acc = \frac{{\sum\limits_{i = 1}^{N} {TP_{i} } + \sum\limits_{i = 1}^{N} {TN_{i} } }}{{\sum\limits_{i = 1}^{N} {TP_{i} } + \sum\limits_{i = 1}^{N} {FP_{i} } + \sum\limits_{i = 1}^{N} {TN_{i} } + \sum\limits_{i = 1}^{N} {FN_{i} } }} \hfill \\ P = \frac{{\sum\limits_{i = 1}^{N} {TP_{i} } }}{{\sum\limits_{i = 1}^{N} {TP_{i} } + \sum\limits_{i = 1}^{N} {FP_{i} } }},R = \frac{{\sum\limits_{i = 1}^{N} {TP_{i} } }}{{\sum\limits_{i = 1}^{N} {TP_{i} } + \sum\limits_{i = 1}^{N} {FN_{i} } }},F1 = \frac{2PR}{{P + R}} \hfill \\ \end{gathered} $$

where $TP_{i} ,FP_{i} ,TN_{i} ,FN_{i}$ denote the numbers of true positive, false positive, true negative and false negative instances in the *i*-th class, respectively, *N* is the number of DDI classes, *F*1 overall measures the precision and recall. The average accuracy loss of the training subset is selected as the model loss rate, the average result on the test set is selected as the model result, and the corresponding iteration time is recorded as the training time of the model.

Hyper-parameter setting for DL models is an open problem. Like the most of hybrid DL models, SCAT has a lot of several hyper-parameters, some key parameters are firstly set as: the number of BiGRU hidden layers, the total iteration times, the learning rate, the dropout rate, and three embedding dimensions of drug sentence, drug description and molecular graph, which are empirically set from Table 3 through their influence on the DDIP performance. The other parameters are randomly initialized as the same as the set in Refs. [12, 16, 20].

**Table 3:** Hyper-Parameter set

Name	Value range	Defulat value
Biomedical word embedding size by BioBERT	100:50:500	400
Drug description embedding size by Doc2Vec	100:40:400	200
Molecule graph embedding size by Graph2Vec	50:20:200	100
Initial learning rate	10^–4^ < *l* < 0.1	0.001
Mini-batch size	10:10:80	50
Number of iterations	1000:100:3000	–
Batch size	–	1024
Drop out	–	0.01
Number of BiGRU layer	1:5	3
Head dimension in Cross-Attention	–	32

**Fig. 5:**
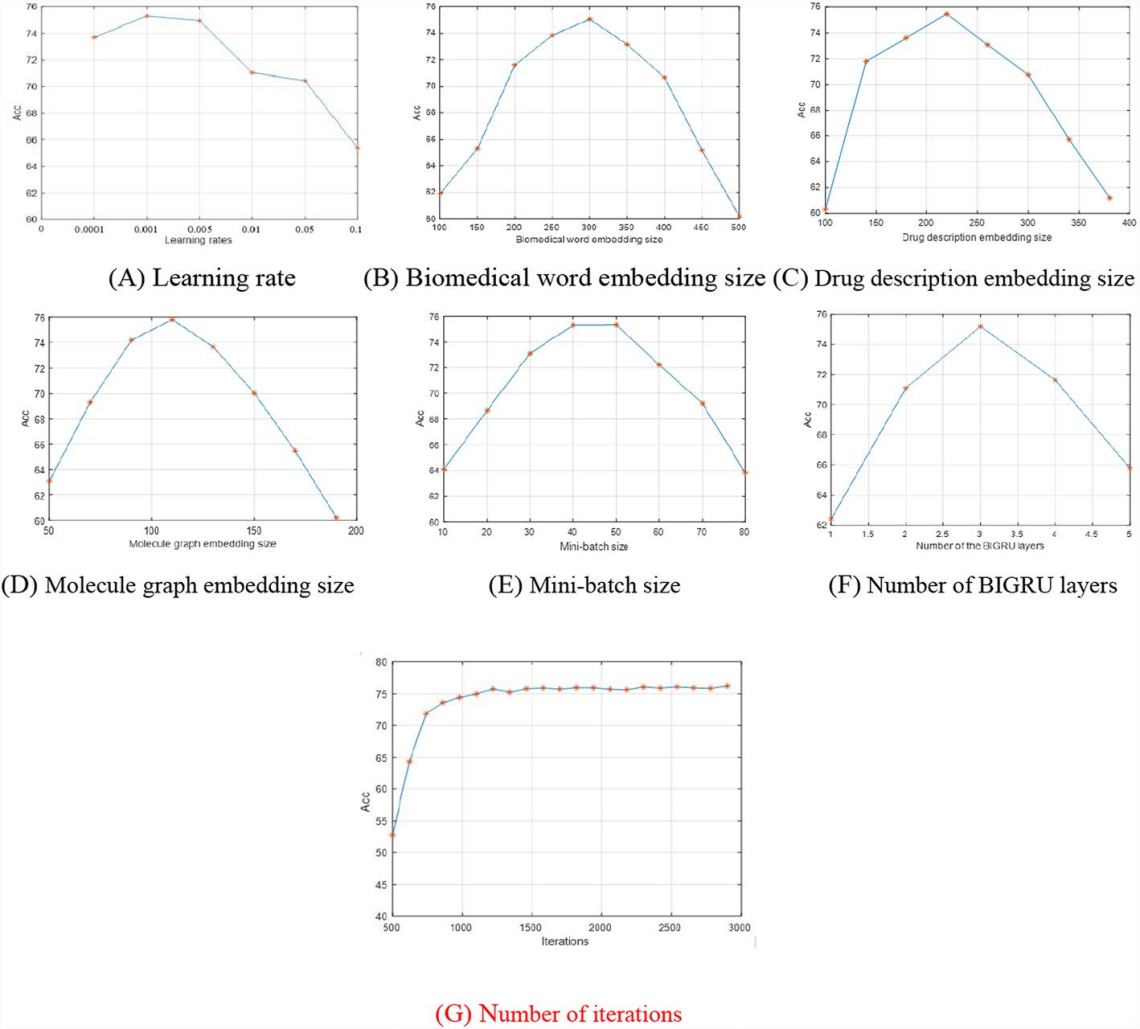
DDIP *Acc* results with different parameters

To discuss the influence of the first 5 parameters on the performance of SCAT, a large number of experiments are conducted on the dataset-4, where one parameter needs to be empirically determined, the other parameters are set to their default values. The results are shown in Fig. 5, where Fig. 5A shows the DDIP *Acc* results versus the learning rate of {0.0001, 0.001, 0.005, 0.01, 0.05, 0.1}, Fig. 5B,C and D show the DDIP *Acc*s with three embedding sizes, 100:50:500, 100:40:400 and 50:20:200, respectively, Fig. 5E shows the DDIP Accs with Mini-batch size 10:10:80, Fig. 5F shows the DDIP Accs with Number of BiGRU layer 1:6, and Fig. 5G shows the DDIP Accs with the number of iterations 1000:100:3000. Each parameter is determined whether the objective function converges to the optimal value and when to converge to the optimal value.

From Fig. 5A to E, it is seen the optimal values of learning rate and Mini-batch size are 0.001 and 30, respectively. From Fig. 5B,C and D, it is found that the appropriate sizes of three embedding dimensions are 400, 200 and 100 for Biomedical word, Drug description and Molecule graph embeddings, respectively. From Fig. 5F, it is seen that the number of BiGRU layers is set as 3. From Fig. 5G, it is obvious that the accuracy increases rapidly before 800 iterations, and becomes stable and converges at about 1500 iterations. To be fair, the number of iterations is set as 3000 for SCAT and the comparison models, i.e., each trained model is determined after 3000 training iterations.

### Experiment results

To compare SCAT with DrugDAGT [16], DDI-GCN [20], DDI-MuG [35], DGNN-DDI [36], and GMIA [37]. Like most of the existing models [10–12, 16, 17, 24], the comparison models are trained with the same hyperparameters settings as SCAT, including learning rate, optimizer, batch size, weight decay, hidden dimension and number of iterations, but are fine-adjusted by 5FCV experiments on four datasets, while their inputs are different from each other. Their results are as shown in Table 4.

**Table 4:** The DDIP results on 4 datasets

Dataset	Method	DrugDAGT	DDI-GCN	DDI-MuG	DGNN-DDI	GMIA	SCAT
	Result (%)						
Dataset-1	Precision	72.72	67.76	69.18	67.61	71.51	75.84
	Recall	70.67	66.66	67.94	65.13	71.88	74.75
	F1	71.68	67.21	68.55	66.35	72.30	75.29
Dataset-2	Precision	72.60	**68.95**	**69.55**	**71.65**	72.75	**76.03**
	Recall	71.42	65.75	67.31	69.72	71.48	73.59
	F1	72.02	67.31	68.41	70.67	72.14	74.79
Dataset-3	Precision	**73.15**	65.60	67.62	65.20	72.86	76.27
	Recall	70.18	63.84	63.85	66.28	71.26	72.43
	F1	71.63	64.71	65.68	65.74	71.22	74.30
Dataset-4	Precision	71.64	65.26	67.16	67.51	**73.06**	75.96
	Recall	71.09	64.80	64.67	67.08	72.82	74.68
	F1	71.36	65.03	65.89	67.29	72.45	75.31

Bold means the best-performing result in a column to improve readability

From Table 4, it is observed that SCAT is superior to the 5 models, followed by DrugDAGT and GMIA. The reason is that SCAT can extract the local and global features and their relationship, and overcome the problem of uneven data samples. GMIA adopts mutual interaction attention mechanism to integrate local and global context between the two associated drug molecules, which can improve DDIP performance. DrugDAGT uses dual-attention graph-Transformer to enable the integration of short- and long-term dependencies within drug molecules. From Table 4, it is obvious that the results of all models on the two original datasets are generally lower than that on the two filtered datasets, because removing the ambiguous DDIs can improve DDIP results. From Table 4, it is also found that the results of all models on the datasets 1 and 2 are better than that on the datasets 3 and 4 without negative instances, because a large number of negative instances can be easily predicted by all models.

**Fig. 6:**
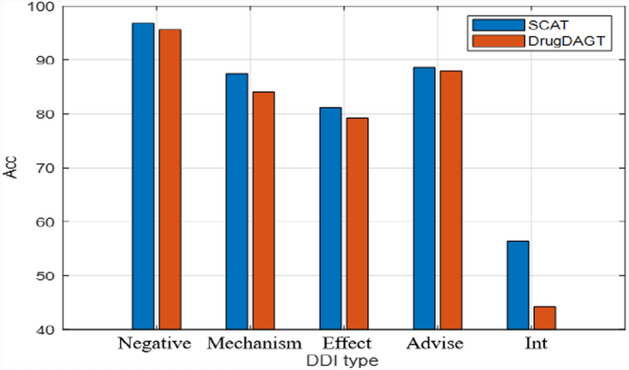
*Acc*s of each DDI type by SCAT and DrugDAGT

To observe the effect of uneven data on SCAT, Fig. 6 shows the *Acc*s of each DDI type on dataset-2 by SCAT and DrugDAGT.

The results in Fig. 6 demonstrate that SCAT is generally superior to DrugDAGT in all DDI types, and increases *Acc* of Negative, Mechanism, Effect and Advise by 1.18%, 3.36%, 1.96%, 0.71% and 12.10%, respectively. SCAT and DrugDAGT get very high *Acc*s on the Negative type because Negative instances dominate the training of the DL model.

### Ablation experiments

Three embeddings of Biomedical word (BW), Drug description (DD) and Molecule graph (MG) are the input data of SCAT. Part-of-speech (POS) features and position information (PI) are two very typical embeddings in NLP tasks. A series of experiments are performed to evaluate the impotance of different embedding features for DDIP. The predicted *Acc*s and training time on dataset-4 are shown in Table 5.

**Table 5:** The DDIP results on the dataset-4 by different embedding features

Result Embedding features	*Acc* (%)	*Training time* (min)
BW	70.14	456
DD	57.18	382
MG	62.23	474
BW + DD	70.46	512
BW + MG	71.37	547
DD + MG	63.28	514
BW + DD + MG	75.46	587
BW + DD + MG + POS	75.51	641
BW + DD + MG + PI	75.49	657
BW + DD + MG + POS + PI	75.52	782

The results in Table 5 indicate that only BW embedding used in the embedding layer achieves a higher Acc of 70.14%, multi-embedding feature fusion can improve DDIP performance, but it will lead to feature redundancy and noise information, and increases the training time. BW of drugs individually achieves an accuracy of 70.14%, which shows that BW is more informative as compared to other features, while DD of drugs individually obtains an accuracy of 57.18%, which show that this feature of drugs is not informative as compared to other. When used individually in combination with other features it produces very good results. MG of drugs achieve accuracy of 62.23%. Whenever using two sets of features, BW + MG achieves an accuracy of 71.37%, which is greater than all of the other drug features used in a set of two. Using three sets of features among all the features, BW + DD + MG achieve an accuracy of 75.46%. Using more sets of features among all the features, BW + DD + MG + POS + PI achieve the highest accuracy of 75.52%, but the training time is much longer. It is also found that BW + DD + MG is the optimal embedding combination, combining POS and PI embeddings slightly improves the model performance, but the training time is much longer.

To further evaluate the effect of different components of SCAT on the DDIP task, a number of experiments are conducted on dataset-4, and the *Acc*s is illustrated in Table 6.

**Table 6:** The *Acc*s by different variants

Result Variant of SCAT	*Acc* (%)
Without BiGRU layer	46.17
Without Cross-Attention layer	62.45
Softmax classifier instead of DDIP layer	71.36

The results in Table 6 validate that the structure of SCAT is optimal, consisting of three main layers: BiGRU, Cross-Attention and DDIP. From Table 6, it is indicated that BiGRU and Cross-Attention are two important modules. The reason is that BiGRU can learn the local–global context features, and Cross-Attention can integrate the relatively more valuable semantic feature and filter noise and capture more semantic feature.

### Result analysis

From the above results, it is known that SCAT achieves remarkable performance on the unbalanced distributed datasets. The main reason is that it can capture the local features and global context information in multimodal data simultaneously, and Cross-Attention can integrate DDIP features, capture the interaction relationship between features, and provide more reliable feature representation. From Table 4, it is found that multimodal data can overcome the sample uneven problem, and the local and global context features extracted from multimodal data are very useful for DDIP task. The second best is DANN-DDI, because it uses multip-drug features and deep attention NN for DDIP. The third best is DrugDAGT, because it can capture short-distance and long-distance dependencies by Transformer. DGNN-DDI is a good interpretation model but with low prediction rate, due to it uses drug chemical structures without other drug information. SCAT is better than DDI-MuG. The reason is that DDI-MuG utilizes the syntactic information within the entire corpus, but without the interaction between the features.

From Table 6, it is observed that using multimodal features of word, drug description and molecule graph can effectively improve the DDIP results, while greatly increasing the training time of the model. From Fig. 6, it is found that category imbalance tends to affect performance of DL in multi-classification tasks. To alleviate class imbalance effect, a simple way is to select roughly the same number of training samples from each DDI type. The above analysis verifies that SCAT can effectively predict potential DDIs.

## Conclusion

Many DDIP methods mainly focus on more powerful feature extraction, while ignoring the related substructure of the drug, and simple connections of drug features fail to capture the complex interactions between drugs. To improve the DDIP performance, a Semantic Cross-Attention Transformer model named SCAT is constructed for biomedical multimodal DDIP. It can fully extract local–global context features and their interactions by BiGRU and Cross-Attention. Specially, its cross-attention module effectively promotes bidirectional feature interactions between drugs, thus extracting more discriminative DDI features. Experimental results on the DDIExtraction2013 dataset demonstrate that SCAT outperforms the most advanced DDIP methods. It is well known that multimodal biomedical data can improve DDIP performance and enhance model interpretability. In future work, due to the complex biomedical corpus and sample imbalance, we will try to use various multimodal data and new embedding approaches to improve DDIP performance and interpretability of SCAT.

## Data Availability

No datasets were generated or analysed during the current study. DDIExtraction2013 (https://pan.baidu.com/s/1CakMXGPqQ4ymwP74phCPQQ Access code: xb9t) is applicable.
